# Heat and Moisture Exchanger Malfunction Causing Dynamic Hyperinflation and Ventilatory Insufficiency Under General Anesthesia: A Case Report

**DOI:** 10.7759/cureus.85192

**Published:** 2025-06-01

**Authors:** Subham Das, Pankaj Deori, Sristi Kumari, Habib Md R Karim

**Affiliations:** 1 Anesthesiology, Critical Care, and Pain Medicine, All India Institute of Medical Sciences, Guwahati, IND

**Keywords:** airway pressure, desaturation, dynamic hyperinflation, humidifier lung, mechanical ventilation

## Abstract

Heat and moisture exchangers (HMEs) humidify inspired gases and preserve airway moisture and temperature. Although considered safe, they can rarely contribute to intraoperative complications. We report the case of a 26-year-old female undergoing diagnostic hysterolaparoscopy under general anesthesia (GA) who developed sudden ventilatory deterioration shortly after endotracheal intubation and initiation of mechanical ventilation. Alarms for high airway pressure and low minute ventilation were triggered, accompanied by rising positive end-expiratory pressure. Initial evaluation, including confirmation of endotracheal tube placement, bronchodilator administration, assessment of ventilator settings, and endotracheal tube adjustment, did not resolve the issue. Re-auscultation revealed diminished breath sounds bilaterally, without any adventitious sounds; manual ventilation also failed to improve ventilation. Further inspection revealed excessive water accumulation in the HME filter within the expiratory limb, despite the HME filter having been in use for the last three hours in a previous case, leading to dynamic hyperinflation due to impaired expiration. Replacement of the clogged filter immediately restored normal ventilatory parameters.

This case highlights a rare but potentially life-threatening complication of HME filter blockage during GA, leading to dynamic hyperinflation. Anesthesia providers should consider circuit-related causes, such as HME malfunction, in the differential diagnosis of acute intraoperative ventilation difficulty. Especially when standard patient and endotracheal tube-related causes are excluded.

## Introduction

Heat and moisture exchangers (HMEs) are frequently used during general anesthesia (GA) to humidify and warm inhaled gases, thereby preserving the thermal and moisture characteristics of the upper respiratory tract [1]. Although they play a crucial role in mechanical ventilation, their use is not without risks [2,3]. Dynamic hyperinflation of the lungs is characterized by inadequate expiration due to airflow limitation. This leads to air trapping, increased end-expiratory volume, and intrinsic positive end-expiratory pressure (PEEP) generation above the extrinsic PEEP applied. This intrinsic PEEP, also known as auto-PEEP, does not allow the lung to deflate well earlier than the extrinsic PEEP level, impairing the exhalation. If expiration is severely impaired, this can result in rapid ventilatory insufficiency [4]. Sudden difficulty in ventilation during GA, accompanied by elevated airway pressures, is a potentially life-threatening event requiring immediate diagnosis and management. While patient-related factors such as bronchospasm, aspiration, and incorrect endotracheal tube placement are usually the first considerations, complications within the airway circuit, including the HME filter, can also contribute to these events [5,6].

Although rare, dynamic hyperinflation during anesthesia may cause significant ventilatory and hemodynamic compromise, potentially culminating in cardiac arrest [7]. We present a case involving a sudden rise in auto-PEEP, increasing inspiratory pressures, and reduced minute ventilation in an otherwise healthy patient shortly after induction of GA and initiation of mechanical ventilation. Pre-anesthesia machine check-up and anesthesia drill to check that the drugs and equipment are routine are standard in our setup. Despite this step, the underlying cause was identified as a water-clogged HME filter connected to the expiratory machine entry point. Informed consent was obtained for publishing the anonymized information.

## Case presentation

A 26-year-old female (55 kg, 165 cm), American Society of Anesthesiologists (ASA) physical status I, with no significant past medical history, was scheduled for diagnostic hysterolaparoscopy for infertility. General anesthesia was induced with IV fentanyl 100 mcg, IV lidocaine 60 mg, IV propofol 110 mg, and IV vecuronium 6 mg. A 7.5 mm cuffed endotracheal tube (ETT) was successfully placed under direct laryngoscopy. Mechanical ventilation was initiated (mode: volume control, tidal volume 8 mL/kg of predicted body weight, respiratory rate 12, inspiratory to expiratory ratio (I:E) of 1:2, PEEP 5 cmH2O) using a standard anesthesia ventilator circuit with an HME filter positioned in the expiratory limb. Sevoflurane was used for maintenance anesthesia, and GA was performed using a Wato-EX 55 Pro anesthesia machine (Mindray Bio-Medical Electronic Co. Ltd., Shenzhen, CHN). 

**Figure 1:**
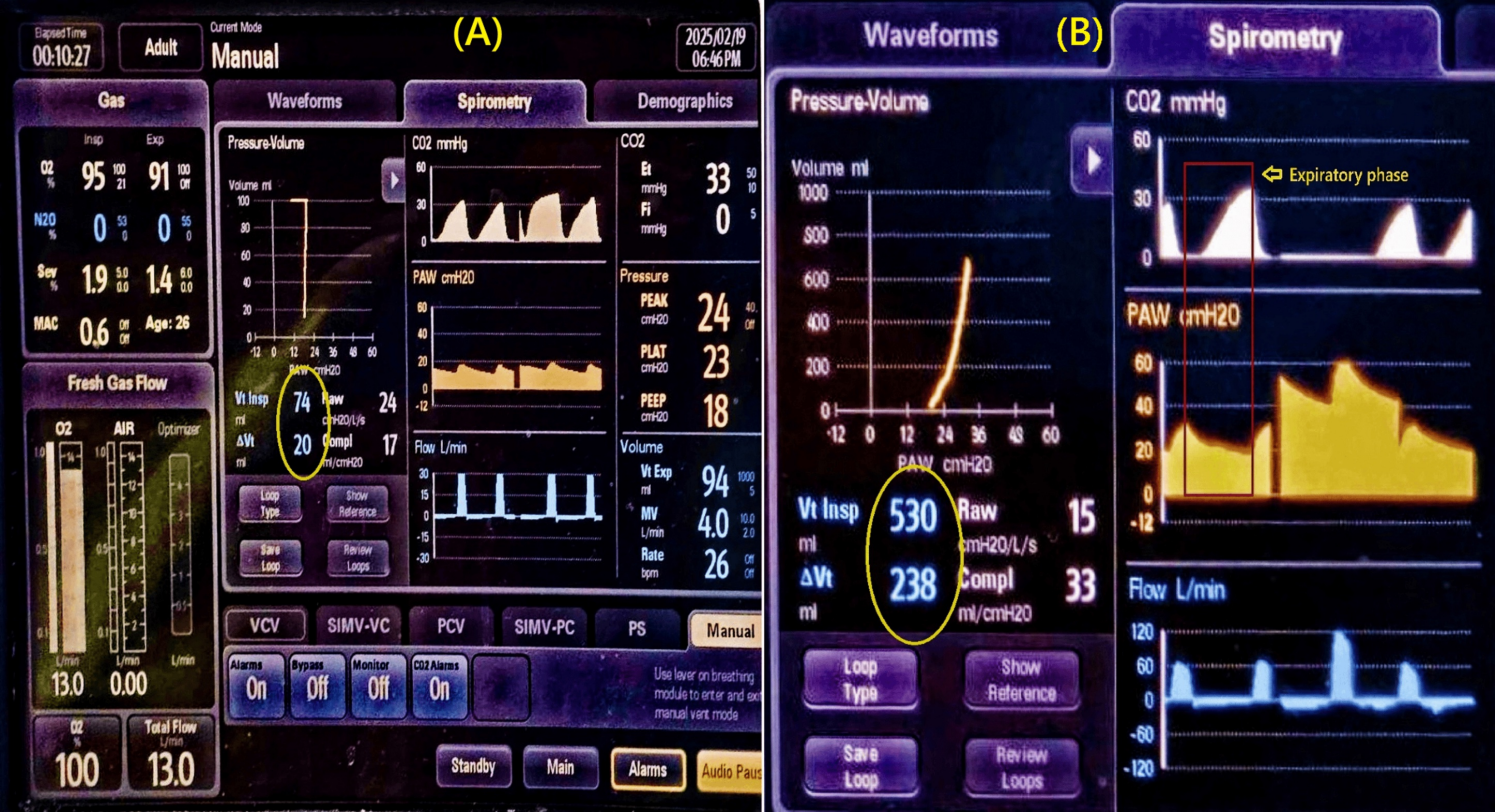
Anesthesia monitor screen (panel A and B) shows decreased return of inspired volume marked within encircle, high PEEP, changing compliance, and expiratory phase resistance shown within red rectangle of panel (B). Vt Insp: Inspired tidal volume, ΔVt: Difference between inspired and expired tidal volume, PEEP: Positive end-expiratory pressure, Raw: Airway resistance

Shortly after initiating mechanical ventilation, the anesthesia machine raised the alarm for high airway pressure and low minute ventilation. Progressive increases in inspiratory pressure reduced tidal volume, and rising PEEP (auto-PEEP) was noted (Figure 1). This persisted even during manual ventilation, with the feel of a tight bag and increased resistance to ventilation.

Standard troubleshooting steps revealed no abnormalities, including confirmation of ETT placement, assessment of ventilator settings, circuit integrity, and auscultation. Oxygen saturation began to decline despite 100% oxygen delivery; hemodynamic parameters were still within an acceptable variation of 20% without any episode of bradycardia. Re-auscultation revealed nearly absent breath sounds bilaterally. Severe bronchospasm was suspected, and salbutamol puffs via ETT and IV hydrocortisone 100 mg were administered. However, ventilation did not improve. Reduced movement of the expiratory valve was noted, and the valve cage was opened, checked, cleaned, and closed. Manual ventilation was attempted without success. Disconnection and brief reconnection of the circuit led to transient improvement, followed by recurrence of the issue.

Further analysis of the expiratory phase and ventilator waveforms revealed signs of expiratory flow limitation and decreasing compliance. Inspection of the HME filter revealed significant water accumulation obstructing airflow (Video 1). Replacement of the clogged HME filter with a new, dry one led to immediate normalization of airway pressures and adequate ventilation. The remainder of the anesthetic course and surgery proceeded uneventfully, with a smooth postoperative recovery.

**Video 1:** Clogged water droplets from condensed exhaled gasses bubbling within the HME HME: Heat and moisture exchanger

## Discussion

This case illustrates an unusual cause of dynamic hyperinflation under GA. While this condition is mostly associated with chronic obstructive pulmonary disease [8], it may rarely occur intraoperatively, even leading to cardiac arrest in extreme cases [9]. Gas trapping was unexpected in our patient, who was previously healthy and ventilated with a standard 1:2 inspiratory-expiratory ratio using protective lung strategies.

Though superior for long-term humidification, heated humidifiers are infrequently used during short-duration GA. However, such practice might vary geographically. Heat and moisture exchangers are more commonly employed for their compact design and ability to filter pathogens [10]. They may be placed at several positions within the breathing circuit, but are often found in the Y-connector and the inspiratory limb. In our case, it was placed near the expiratory port of the machine end, which led to the issues confined to the expiratory phase only. Such water-clogging in the HME filter and malfunction is expected to cause an issue with both the inspiration and expiration phases when placed in the Y-connector and ETT or cath-mount. 

**Figure 2:**
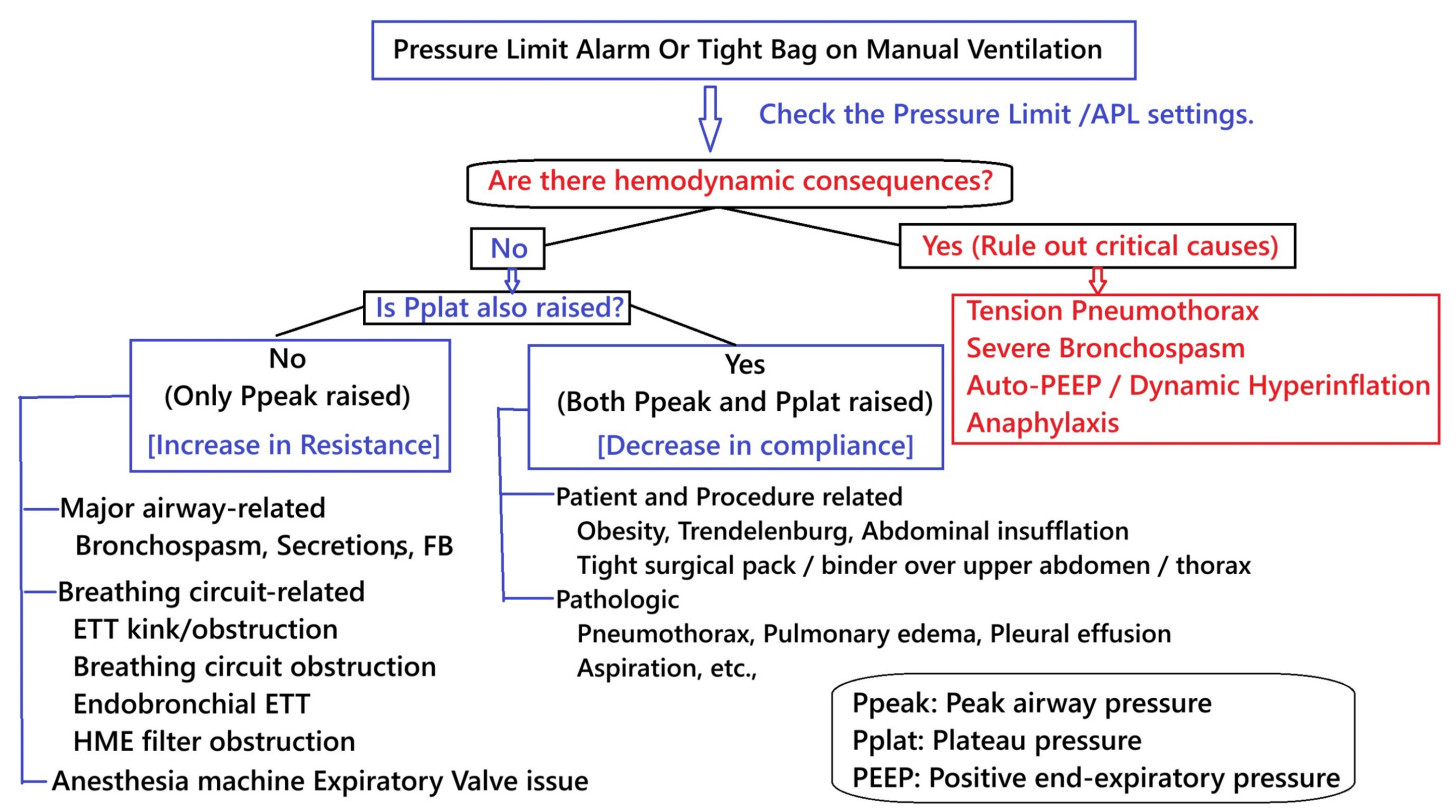
An approach on how to address intraoperative raised airway pressure alarm or tight bag APL: Adjustable pressure limiting, ETT: Endotracheal tube, FB: Foreign body, HME: Heat and moisture exchanger, Pplat: Plateau pressure, Ppeak: Peak airway pressure, PEEP: Positive end-expiratory pressure

Despite their benefits, HMEs can introduce complications: disconnection, leaks, and, in rare cases, blockages from moisture accumulation [1,11]. In this case, water vapor from exhaled gases condensed within the HME's electrostatic membrane, leading to progressive obstruction. Many HME filters are hygroscopic and comprise papers and foams embedded within a hygroscopic salt, which absorbs and exchanges heat and moisture. While HME blockage is an uncommon cause of intraoperative ventilatory failure, it should remain on the differential, especially when patient-related factors have been ruled out. The common causes and possible flow for finding the causes of a raised pressure alarm or a tight bag on manual ventilation are presented in Figure 2. 

Other possible diagnoses, such as bronchospasm, anaphylaxis, mucus plugging, pneumothorax, and endotracheal tube misplacement, were considered and systematically excluded through clinical evaluation and therapeutic trials. The absence of skin manifestations or hemodynamic instability made anaphylaxis unlikely, and no improvement was noted with bronchodilators or suctioning. Ventilator dyssynchrony was ruled out by ensuring complete muscle relaxation.

We perform routine comprehensive pre-anesthesia machine checkups as per standard prescribed recommendations [12]. The practice of HME filter change might vary. Evidence even shows that an HME filter can be used up to 48 hours without increasing the risk for pneumonia [13]. However, such use is in the same patient, and practice might vary in the operating theater. We usually do not replace the HME filter for each case unless we perform GA on a patient with known or suspected respiratory disease with possible droplet transmission, or it's visibly soiled. Further, we need to consider the type of HME used, as hygroscopic filters absorb and retain water vapor, which leads to swelling of the paper or foam component and can cause obstruction to the flow. This case reinforces the importance of inspecting external components of the breathing system when unexplained ventilatory deterioration occurs. Notably, HMEs are expected to provide 90% to 95% relative humidity, though most achieve ~70% [14]. Prolonged use, particularly in high-humidity environments like the operating room, can predispose to water accumulation and filter obstruction [15]. The HME filter placement above the lung level and regular monitoring may help mitigate this risk.

## Conclusions

An HME filter obstruction is a rare yet significant cause of intraoperative ventilatory compromise and dynamic hyperinflation. When unexplained rises in airway pressure and reductions in tidal volume occur, a high index of suspicion is warranted. Prompt recognition, aided by ventilator graphics and clinical vigilance, is critical for effective management. While internal (patient-related) causes are often prioritized, malfunction of external devices such as HME filters should not be overlooked. Our observation is, however, limited to a single case, and the HME filter is placed in the expiratory limb. Placement at other places is expected to lead to different respiratory dynamics.
